# S170. AMYGDALA SUBNUCLEI VOLUMES IN FIRST-EPISODE PSYCHOSIS: ASSOCIATION WITH CHILDHOOD ADVERSITY

**DOI:** 10.1093/schbul/sby018.957

**Published:** 2018-04-01

**Authors:** Reetta-Liina Armio, Heikki Laurikainen, Raimo Salokangas, Lauri Tuominen, Jarmo Hietala

**Affiliations:** 1 University of Turku; 2 Harvard University, University of Turku

## Abstract

**Background:**

The amygdala volume is reduced already in the first episode of psychosis. The amygdala is a key region in emotional processing, and its volume reduction has been associated with severity of childhood adversity in psychotic patients. Since the amygdala is comprised of separate subnuclei with distinct anatomy and function we wanted to study whether these effects are present in some subnuclei more than others in first episode of psychosis.

**Methods:**

We studied amygdala subnuclei volumes in 68 first-episode psychosis (FEP) patients (mean age = 27.1 ± 6.2, 35 females) and 65 healthy controls (mean 28.9 ± 6.5, 33 females) randomly selected from the general population. Subjects underwent a T1-weighted MRI with 1mm isotropic resolution (Philips Ingenuity 3T). The subnuclei volumes were generated with a new automated algorithm in FreeSurfer. Childhood adversity was measured using the Trauma and Distress Scale Scores (TADS). Baseline group differences in the amygdala subnuclei volumes were tested using repeated measures general linear model. The analyses were restricted to the four largest subnuclei: the lateral, basal, accessory basal, and the corticoamygdaloid transition area with volumes > 100 mm3. There were no differences between hemispheres nor group by hemisphere interactions so left and right hemispheres were averaged. All group comparisons were corrected for age, sex, and total intracranial volume. Association between the volumes and the TADS scores in the FEP group were also corrected for cumulative exposure to antipsychotic medication.

**Results:**

We found that amygdala subnuclei were smaller in the FEP patients than in the the controls with regional specifity (subnucleus ROI*Group p = 0.015). In the FEP, the most robust reductions were in the lateral nucleus (Bonferroni corrected p = 0.036, β = -64.15). No statistically significant difference was observed in the basal nucleus, the accessory basal nucleus or the corticoamygdaloid transition area. The FEP patients had in average higher TADS total score (19.00 ± 13.56) compared to the HC (7.68 ± 7.07) (p < 0.001, t = 5.84).

We found that particularly the TADS physical abuse score (FEP(n)=63, HC(n)=59) associated significantly differently with some subnuclei in patients and control group (ROI*Group*Physical abuse p = 0.016). The difference was significant only in the lateral nucleus (Group*Physical abuse p = 0.048, β = -34.97). However, there was an overall nonsignificant trend of the negative association between lateral nucleus volume and all TADS scores in the FEP. Similar trend was not seen in the controls.

**Discussion:**

We show that the amygdala subnuclei are differently affected already in the first episode of psychosis. Compared to the controls, the FEP patients had smaller lateral nucleus volume, but not basal, accessory basal nucleus or corticoamygdaloid transition area. The lateral nucleus volume was also negatively associated with childhood traumatic experiences, particularly physical abuse in the FEP patients. These findings suggest the involvement of the lateral nucleus of amygdala in the association between childhood traumatic experiences and psychotic disorders. This is well in agreement with studies suggesting that the lateral nucleus of the amygdala is associated with fear learning, recovery from fear and regulation of fear expression.

